# Thromboembolic and Bleeding Risk in Atrial Fibrillation Patients with Chronic Kidney Disease: Role of Anticoagulation Therapy

**DOI:** 10.3390/jcm10010083

**Published:** 2020-12-28

**Authors:** Michele Magnocavallo, Antonio Bellasi, Marco Valerio Mariani, Maria Fusaro, Maura Ravera, Ernesto Paoletti, Biagio Di Iorio, Vincenzo Barbera, Domenico Giovanni Della Rocca, Roberto Palumbo, Paolo Severino, Carlo Lavalle, Luca Di Lullo

**Affiliations:** 1 Department of Clinical, Internal, Anesthesiology and Cardiovascular Sciences, Policlinico Universitario Umberto I, Sapienza University of Rome, 00161 Rome, Italy; michelefg91@gmail.com (M.M.); marcoval.mariani@gmail.com (M.V.M.); paolo.severino@uniroma1.it (P.S.); carlolavalle@yaoo.it (C.L.); 2 Department of Research, Innovation and Brand Reputation, ASST-Papa Giovanni XXIII, 24127 Bergamo, Italy; antoniobellasi@hotmail.com; 3 National Council of Research, Institute of Clinical Physiology, 56124 Pisa, Italy; dante.lucia11@gmail.com; 4 Nefrologia, Dialisi e Trapianto, Policlinico San Martino, 16132 Genova, Italy; maura.ravera@hsanmartino.it (M.R.); ernesto.paoletti@hsanmartino.it (E.P.); 5 Department of Nephrology and Dialysis, Moscati Hospital, 83100 Avellino, Italy; brdiiorio@gmail.com; 6 Department of Nephrology and Dialysis, Parodi-Delfino Hospital, 00034 Colleferro, Italy; vincenzobarbera58@gmail.com; 7 Texas Cardiac Arrhythmia Institute, St. David’s Medical Center, Austin, TX 78705, USA; domenicodellarocca@hotmail.it; 8 Department of Nephrology and Dialysis, Sant’Eugenio Hospital, 00144 Rome, Italy; palumbo.dr@gmail.com

**Keywords:** atrial fibrillation, chronic kidney disease, warfarin, direct oral anticoagulants, end stage renal disease, left atrial appendage occlusion

## Abstract

Atrial fibrillation (AF) and chronic kidney disease (CKD) are strictly related; several independent risk factors of AF are often frequent in CKD patients. AF prevalence is very common among these patients, ranging between 15% and 20% in advanced stages of CKD. Moreover, the results of several studies showed that AF patients with end stage renal disease (ESRD) have a higher mortality rate than patients with preserved renal function due to an increased incidence of stroke and an unpredicted elevated hemorrhagic risk. Direct oral anticoagulants (DOACs) are currently contraindicated in patients with ESRD and vitamin K antagonists (VKAs), remaining the only drugs allowed, although they show numerous critical issues such as a narrow therapeutic window, increased tissue calcification and an unfavorable risk/benefit ratio with low stroke prevention effect and augmented risk of major bleeding. The purpose of this review is to shed light on the applications of DOAC therapy in CKD patients, especially in ESRD patients.

## 1. Introduction

**Figure 1:**
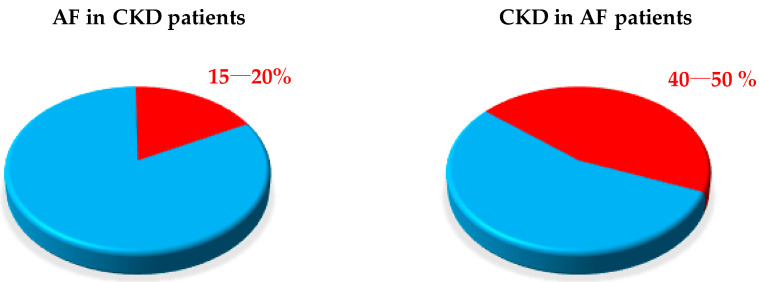
Prevalence of atrial fibrillation (AF) in chronic kidney disease (CKD) patients and vice versa.

The prevalence of atrial fibrillation (AF) in the general population ranges between 0.5% and 1%, with peaks of 8% in patients over 80 years of age [1]. AF patients are at increased risk of thromboembolic complications, and oral anticoagulant therapy is universally recommended in clinical guidelines [2,3,4,5,6,7]. Chronic kidney disease (CKD) is also associated with increased cardiovascular disease risk and all-cause mortality [8,9,10] and is highly prevalent in the AF population, affecting 40–50% of patients with AF (Figure 1) [11,12,13].

Similarly, AF coexists in up to 15–20% of CKD subjects, especially in end stage renal disease (ESRD) patients, who are identified on the basis of an estimated glomerular filtration rate (eGFR) < 15 mL/min, including those requiring dialysis (Table 1) [2,13,14,15,16].

Furthermore, patients with advanced CKD (eGFR < 30 mL/min) are at increased risk of bleeding from uremia-induced platelet dysfunction and invasive procedures related to dialysis [17,18,19].

Randomized controlled trials demonstrated that direct oral anticoagulants (DOACs) are not inferior to warfarin for stroke or systemic embolism; however, these studies excluded patients on dialysis, those with an eGFR < 25–30 mL/min and those treated with vitamin K antagonists (VKA) other than warfarin [20,21,22,23,24,25]. Consequently, all data concerning use of DOACs in patients with eGFR < 30 mL/min came from observational studies, and the evidence in favor of DOACs in patients with advanced or ESRD is still very limited [26,27,28,29,30,31]. The aim of this review is to evaluate how treatment with DOACs affects stroke and bleeding outcomes compared with warfarin in a CKD population. Moreover, particular consideration is given to the role of long-term oral anticoagulant therapy in renal preservation function.

## 2. Pathophysiology of High Thromboembolic/Hemorrhagic Risk in CKD Patients

AF and CKD are strictly related and share several risk factors (hypertension, diabetes, obesity, metabolic syndrome). Consequently, the growing incidence and prevalence of AF are linked with a parallel rise in CKD and vice versa [32,33]. Furthermore, progressive worsening of kidney function is associated with an increased rate of AF, and in dialysis patients, prevalence of AF reaches about 16% [12,16,34]. Contemporary presence of AF and CKD outlines a clinical condition characterized by a very high thromboembolic risk (cardioembolic stroke, systemic thromboembolic and death) and unexpected elevated hemorrhagic risk, especially in dialysis patients.

The central role of CKD in raised thromboembolic risk is well known. Piccini et al. have demonstrated that impaired renal function is a great predictor of cardioembolic stroke and systemic embolism [35]. Therefore, for a better evaluation of thromboembolic risk, they have proposed to extend the CHADS_2_ score with an additional 2 points for patients with eGFR < 60 mL/min, the so-called R_2_CHADS_2_ score [35].

**Figure 2:**
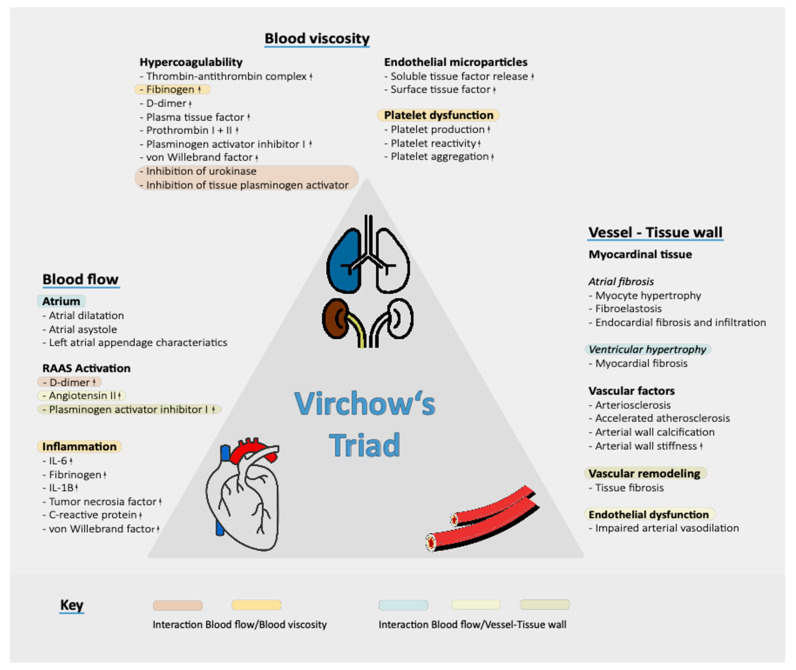
Factors increasing the propensity of thrombus formation in CKD patients.

Several factors increase the propensity of thrombus formation in patients with CKD; as depicted in Figure 2, all Virchow’s triad elements (abnormalities in blood flow, vessel wall and blood constituents) appear abnormal. Additionally, reduced eGFR is an independent predictor of low left atrial appendage contractility and emptying velocity [36,37]. These elements promote the formation in the left atrium of dense spontaneous echocardiographic contrast, which is an indicator of relevant blood stasis and is associated with augmented thrombogenic risk [38,39].

On the other hand, CKD patients have an increased atherosclerosis susceptibility with a bigger pulse wave velocity and reduced flow-mediated endothelium-dependent dilation [40,41]. Higher endogenous levels of Endotelin-1 and plasma cAMP in CKD individuals seem to be associated with an increased thromboembolic susceptibility [42].

Lastly, CKD is associated with an increase of inflammatory and procoagulant biomarkers that enhance platelet activity and clot formation [43,44]. Reduced metabolism of C-reactive protein, anomalous expression of glycoprotein Ib, increased levels of pro-inflammatory proteins (IL-1, TNF alfa, D-Dimer) and procoagulant factors (VII, VIII, fibrinogen, Von Willebrand, plasminogen activator inhibitor-1) and inhibition of plasmin by increased levels of lipoprotein(a) are the most important hematological abnormalities described in CKD patients [45,46,47,48].

**Figure 3:**
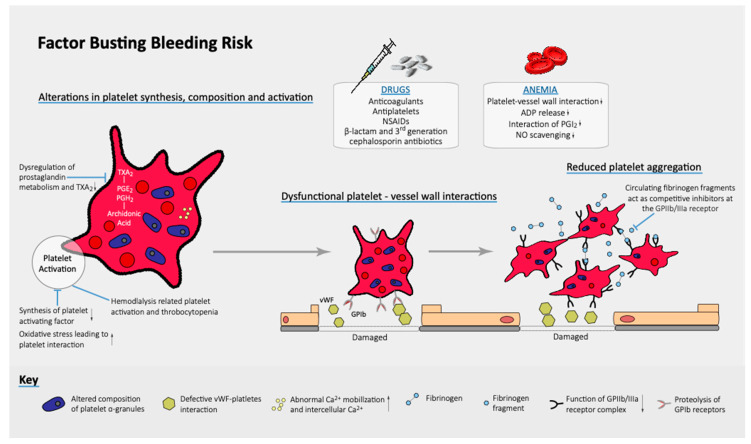
Factors contributing to pro-hemorrhagic state in CKD patients.

Such factors are also involved in an augmented hemorrhagic risk. Specifically, platelet abnormalities, uremic toxins, uncontrolled hypertension, repeated cannulations for dialysis and invasive procedures contribute to a remarkably high risk of bleeding (Figure 3). Above all, platelet disfunctions seem to be predominant and include reduction in intracellular ADP, impaired release of the platelet alpha-granule protein, enhanced intracellular cAMP, anomalous arachidonic acid metabolism and cyclo-oxygenase activity, aberration of the activity of GP IIb/IIIa and altered von Willebrand factor promoting a pro-hemorrhagic state [49,50,51]. Moreover, uremic toxins alter blood flow and enhance erythropoietin deficiency [51,52].

Based on previous evidence that proved the high thromboembolic/hemorrhagic risk in CKD patients, it is conceivable that a new risk chart, specifically constructed for renal patients, may improve risk stratification of both thromboembolic and hemorrhagic events [53].

## 3. Anticoagulant-Related Nephropathy and Progression of Kidney Disease

Despite increasing use of oral anticoagulants in the last 20 years, only in 2009 did Brodsky et al. introduce the concept of “warfarin-related nephropathy” (WRN). WRN is a particular form of acute kidney injury (AKI) without any obvious underlying cause, in a patient treated with warfarin with an international normalized ratio (INR) > 3.0 and microscopic or gross hematuria [54]. Brodsky et al. performed renal biopsies in nine patients with unexplained AKI and supratherapeutic INR; histological specimens showed a pattern of diffuse dysmorphic erythrocyte accumulation both in kidney tubules, some of which appeared obstructed and dilated, and in the glomerulus, especially in Bowman’s space [54]. The two main pathophysiological processes to explain AKI are the disruption of the glomerular filtration barrier causing bleeding into Bowman’s space and the aggregation of red blood cells, forming casts in the tubules, which lead to their obstruction and ischemia [54]. Supratherapeutic anticoagulation seems to play an essential role in inducing WRN, but it is likely that a second factor is required; a considerably reduced number of nephrons or acute damage to glomeruli seems to be the conditions contributing to glomerular bleeding in case of over-anticoagulation. Causes of acute nephron damage could be congestive heart failure, recent initiation of renin–angiotensin system inhibitors, thromboembolic kidney disease, endocapillary proliferative or crescentic glomerulonephritis or bladder clots causing ureteral obstruction. In a patient–control study enrolling 15,258 patients who initiated warfarin during a 5-year period, a presumptive diagnosis of WRN occurred in 20.5% of the entire cohort and in 33.0% of the CKD cohort [55]. The 1-year mortality in patients experiencing WRN was 31.1% compared with 18.9% in patients without WRN, which represents an increased risk of 65% [55]. Overall, WRN may be considered not only a common complication of VKA therapy but also a powerful negative prognostic factor.

Since 2009, several studies have confirmed the hypothesis proposed by Brodsky that excessive anticoagulation is associated with WRN [56,57,58,59]. Golbin et al. described the largest biopsy-proven case series of AKI induced by other VKAs, specifically the first cases of AKI by fluindione and acenocoumarol [60]. Of note, no clinical or histological differences were reported in patients treated with warfarin or fluindione/acenocoumarol [60].

**Figure 4:**
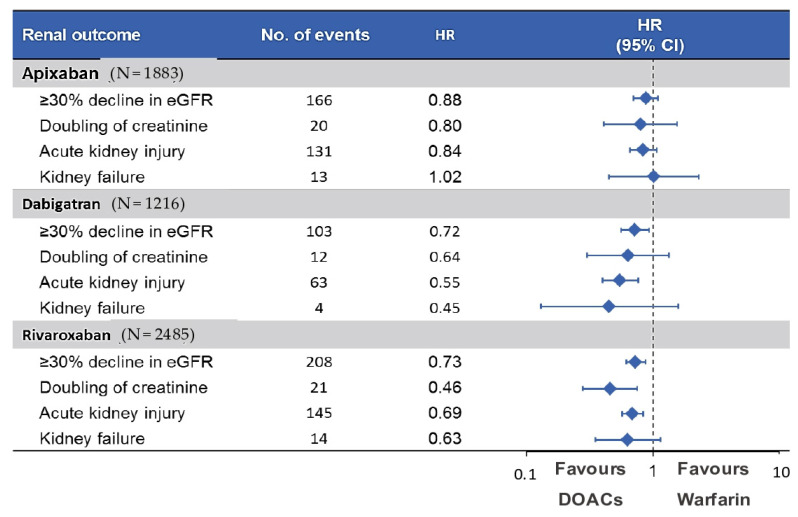
Comparison between direct oral anticoagulants (DOACs) and warfarin in terms of renal preservation.

**Figure 5:**
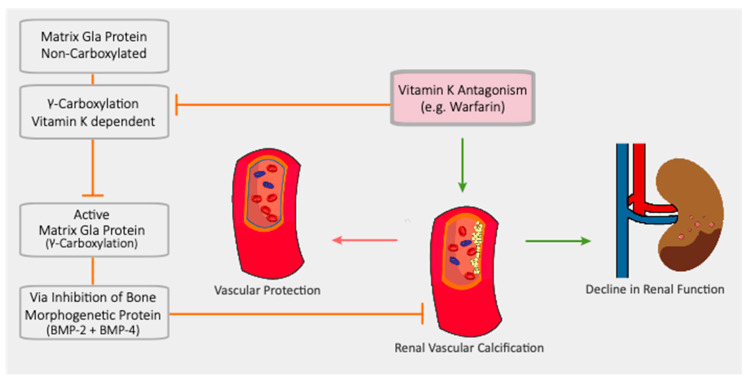
Vascular calcification, arterial and renal damage induced by inhibition of vitamin-K-dependent MGP.

The connection between AKI and anticoagulation has also been extended to DOACs; therefore, the term WRN was gradually replaced by the more inclusive “anticoagulant-related nephropathy” (ARN) [61,62,63,64]. Given the paucity of renal outcomes reported in studies involving DOACs and the lack of limited long-term data, it is possible that the true incidence of ARN is under-recognized. Two large retrospective studies demonstrated that apixaban, dabigatran and rivaroxaban are associated with a lower risk of AKI compared to warfarin (Figure 4) [26,65]. Overall, VKA administration is still considered a major risk factor for AKI, as a result of vascular calcification due to inhibition of the vitamin-K-dependent matrix gamma-carboxyglutamate protein (MGP), as depicted in Figure 5 [66,67,68,69,70]. Similar findings were also reported in a cohort of AF patients undergoing percutaneous coronary intervention; after administration of contrast medium, patients taking DOACs, especially dabigatran, showed a better control of renal function than patients on warfarin with a trend toward a reduction in the incidence of AKI [71].

Although the new European Society of Cardiology guidelines for AF recommend the use of DOACs for long-term oral anticoagulation, and the previous observational studies have showed how these drugs should play an important role in the preservation of renal function, a large study comparing DOACs across different stages of kidney function revealed that the proportion of patients using DOACs decreases in parallel to the decreasing kidney function [72]. Indeed, in patients with eGFR ≥ 90 mL/min, a DOAC was prescribed in 73.5% of cases, while in patients with eGFR between 15 and 30 mL/min, a DOAC was prescribed in only 45.0% of cases [72]. Notably, no difference in terms of mortality was reported among the three DOACs, and each one consistently showed at least equivalent effectiveness and safety compared with warfarin across the range of kidney functional stages, confirming the promising findings in this particular patient setting [72].

In conclusion, progression of kidney failure represents a central issue in the management of long-term oral anticoagulation, especially in elderly patients in which AF and CKD coexist in up to 25% of cases [13,34]. AF can deteriorate renal function over time, and eGFR worsening is an independent predictor of ischemic stroke/systemic embolism [73,74,75]. In these high thromboembolic and hemorrhagic risk patients, renal function should be regularly monitored, preferably after 1 month initially and at least every 3 months thereafter [3].

## 4. DOACs, Diabetes and Chronic Kidney Disease

**Figure 6:**
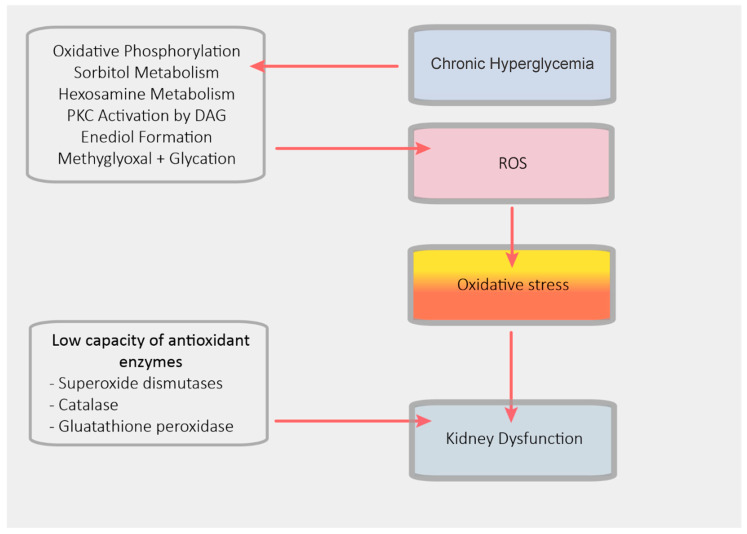
Pathophysiology of diabetic kidney disease. DAG: diacylglycerol; PKC: protein kinase C; ROS: reactive oxygen species.

With regard to the progression of CKD, it is crucial to highlight the close relationship between AF, diabetes mellitus (DM) and CKD; nearly 25% of patients with CKD are also diabetic [76]. As described in Figure 6, microvascular complications in DM could worsen kidney function and contribute to the onset of diabetic kidney disease (DKD), which affects about one-third of DM patients [77,78,79,80]. Long-term thromboembolic preventive therapy in AF patients with DM and CKD may be more challenging because both DM and CKD have been independently associated with an increased thromboembolic and bleeding risk, which results from the prothrombotic and pro-inflammatory status [81,82,83,84,85]. In diabetic patients, metabolic abnormalities predispose arteries to atherosclerosis and increase platelet reactivity and blood coagulability [80,81,82,86,87]. Simultaneously, progressive worsening of kidney function is associated with an increased rate of AF and a major bleeding risk [17,34].

Emerging data suggest that DOACs may be associated with better preservation of renal function when compared to warfarin [55,59,88,89]. As previously described, VKAs may also induce renal damage due to increased vascular calcification resulting from vitamin-K-dependent MGP inhibition [66,67,69]. In a study by Fusaro et al., MGP seemed to be reduced in patients affected by DM and CKD, predisposing them to a worse renal outcome when treated with VKA [90,91,92,93,94]. In contrast, rivaroxaban may provide renal preservation by decreasing vascular inflammation through reducing PAR-1 and PAR-2 signaling [95]. AF diabetic patients treated with rivaroxaban showed a lower incidence rate of hospitalization for AKI, progression to stage 5 CKD or hemodialysis than patients treated with warfarin [95]. Furthermore, in the post-hoc ROCKET AF analysis, rivaroxaban showed consistently better safety and efficacy compared to warfarin in AF patients with DM [96].

Real-world evidence supports the findings that renal function is better maintained in DM patients receiving DOACs rather than warfarin. A subgroup analysis of the RELOAD study investigated the effectiveness and safety of rivaroxaban versus warfarin in patients with AF and DM; risk of AKI and ESRD were decreased in diabetics taking rivaroxaban [95].

**Figure 7:**
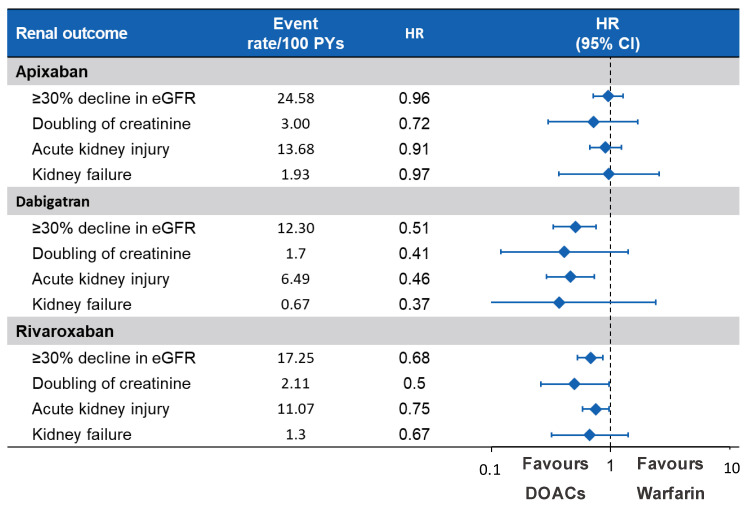
Comparison between DOACs and Warfarin in terms of renal preservation in diabetic patients.

In an analysis performed by Yao W et al. on a large heterogeneous cohort of AF patients with diabetes (Figure 7), treatment with DOACs was related to lower incidence of worsening renal function, defined as a ≥30% decline in eGFR, doubling of serum creatinine or AKI [26].

According to the latest evidence, we consider DOACs more effective and safer than warfarin for prevention and progression of kidney disease in AF patients with diabetes.

## 5. DOACs and End Stage Renal Disease

The increased hemorrhagic risk and the lack of safe evidence for an effective risk/benefit ratio are the principal reasons for the restricted use of anticoagulants in CKD patients, especially those undergoing renal replacement therapy (RRT) [97]. In RRT patients, considering that the elimination of drugs is strictly dependent on the size of the molecules, the percentages linked to plasma proteins and the physicochemical properties of the dialysis filter, warfarin and DOACs are both poorly eliminated by dialysis clearance. While the superiority of DOACs vs. warfarin is well documented in patients with preserved renal function or moderate CKD, there is a lack of currently available data for DOACs in patients with severe CKD or ESRD that may lead to an increased risk of bleeding [25]. Indeed, there are no randomized controlled trial data on the use of DOACs for stroke prevention in AF patients with severe CKD or on RRT, since all landmark DOAC trials excluded patients with eGFR < 30 mL/min (except for a few patients on apixaban with eGFR 25–30 mL/min) [20,21,22,23,24].

The main data on the use of DOACs in RRT patients are from studies in the USA. Dabigatran 110 or 150 mg twice daily resulted in a higher exposure compared with standard RE-LY patients (1.5- to 3.3-fold increase in area under the curve); dabigatran 75 or 110 mg once daily produced exposures comparable to those simulated in typical RE-LY patients. These data appear to suggest that the reduced dose regimen may be more suitable for hemodialysis patients [23,98]. More detailed information is available about Apixaban’s pharmacokinetic characteristics. ESRD resulted in a modest increase (36%) in apixaban area under the curve with no increase in its peak concentration [99]. Apixaban 2.5 mg b/die administered to hemodialysis patients resulted in a drug exposure similar to that of the standard dose (5 mg b/die) in patients with preserved renal function, while apixaban 5 mg twice daily is associated with supratherapeutic levels in ESRD [100]. Moreover, apixaban is highly protein bound, and in case of a bleeding event, reversal of the anticoagulant activity with prothrombin complex concentrate should be attempted instead of dialysis.

Similar findings were reported with rivaroxaban 10 mg/die in hemodialysis patients as compared to the standard dose (20 mg/die) in patients with normal kidney function [101]. Surprisingly, deterioration of renal function from severe to ESRD does not seem to have a significant impact on the rivaroxaban pharmacokinetic and anticoagulation effect compared with those changes observed with either moderate or severe renal impairment [102].

**Figure 8:**
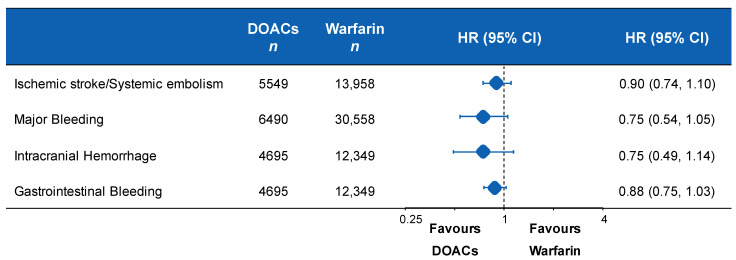
DOACs vs. warfarin in non-valvular AF patients with advanced kidney disease or undergoing dialysis.

Although current data on the efficacy and safety of DOACs in ESRD are limited, they are very encouraging (Figure 8) [103].

In a retrospective cohort study, apixaban was superior in ESRD patients in terms of both safety and effectiveness when compared with warfarin; both the standard (5 mg/bd) and the reduced (2.5 mg/bd) doses of apixaban were associated with lower major bleeding risks, but only the standard dose was associated with reduced thromboembolic events and mortality [30]. Miao B et al. compared rivaroxaban and apixaban in ESRD patients. No significant differences were reported in terms of thromboembolic and hemorrhagic risk [31]; however, when compared to warfarin, rivaroxaban appears to be associated with a reduction of major bleeding [104]. Furthermore, a meta-analysis enrolling 71,877 patients on long-term dialysis and with AF showed that patients receiving apixaban 5 mg twice daily had a significantly lower risk of mortality than those receiving apixaban 2.5 mg twice daily, warfarin or no anticoagulant and lower bleeding risk than those on warfarin, dabigatran or rivaroxaban [105]. Overall, among patients with advanced CKD and ESRD, the use of apixaban was associated with lower risk of major bleeding compared to warfarin and was effective in preventing systemic embolism [106].

To date, only rivaroxaban 15 mg/die and apixaban 5 mg/bd (reduced dose 2.5 mg/bd in patients 80 years or older weighing 60 kg or less) are approved by the Food and Drug Administration as a long-term oral anticoagulant in ESRD patients. Despite the mounting evidence about the possibility of using DOACs in patients with eGFR < 15 mL/min, the nephrological guidelines KDIGO (Kidney Disease: Improving Global Outcomes) still recommend warfarin as the first choice drug and suggest the possibility of percutaneous or surgical closure of the left atrial appendage [107]. A randomized trial comparing DOACs and warfarin in ESRD patients might be appropriate for clarifying which is the safest and most efficient long term stroke prevention therapy in ESRD and AF patients. Randomized controlled trials are underway comparing DOACs with warfarin in advanced CKD or dialysis patients. The AXADIA study (Compare Apixaban and Vitamin-K Antagonists in Patients with Atrial Fibrillation and End-Stage Kidney Disease) is randomizing patients to apixaban 2.5 mg/bd or phenprocoumon individually adjusted to an INR of 2.0–3.0; the study completion date is scheduled for July 2023 (NCT02933697) [108]. Similar rates of major and clinically relevant non-major bleeding events were reported in the RENAL-AF trial in which patients were randomized to apixaban 5 mg/bd or warfarin (NCT02942407). Unfortunately, the study was stopped early and enrolled only 154 of the 762 expected patients, so the small sample size and low event rate are significant limitations of the study.

## 6. Non-Anticoagulative Approaches

Patients with ESRD represent the most complex population for long-life anticoagulant management. In the current European Guidelines, DOACs are contraindicated in patients with eGFR < 15 mL/min (ESRD), and VKAs remain the only drugs allowed [3]. Phenprocoumon and acenocoumarol have more advantageous pharmacokinetic properties than warfarin. Acenocoumarol has a shorter half-life, while the effects of CYP2C9 polymorphisms are least pronounced in the case of phenprocoumon [109,110]. On the other hand, warfarin has a narrow therapeutic window and several drug–drug and drug–food interactions; moreover, it seems to increase tissue calcification, including cardiac valves, and precipitate calcific uremic arteriolopathy [111,112,113]. For these reasons, patients treated with phenprocoumon and acenocoumarol require fewer monitoring visits than those prescribed warfarin. Nevertheless, the therapeutic range for all VKA drugs is usually unsatisfactory; as a consequence, thromboembolic and hemorrhagic events are more frequent in patients treated with VKAs than DOACs [112,114,115,116,117]. Lastly, although treatment adherence was comparable between DOACs and VKAs, treatment satisfaction and persistence are significantly lower with VKAs than DOACs; CKD and history of bleeding represent some of the main factors associated with absence and/or non-adherence to anticoagulant therapy in everyday practice [97,118,119,120].

Percutaneous left atrial appendage occlusion (LAAO) has emerged as a potential alternative to life-long oral anticoagulation because 90% or more of thrombi during AF are located in the left atrial appendage, a remnant of the primordial left atrium [121]. This strategy is currently limited to patients with a high thromboembolic and bleeding risk who are ineligible for long term OACs. Based on the available data, the use of LAAO will likely grow tremendously in the next few years because the periprocedural major adverse event rate is very low in patients with several comorbidities and high thromboembolic/hemorrhagic risk [122,123,124,125,126,127,128,129].

In patients with advanced CKD, percutaneous LAAO appears to have a similar risk of periprocedural complications compared to patients without significant renal impairment [130,131].

Additionally, recent studies have explored its efficacy for thromboembolic prevention in patients with end-stage renal disease [131,132,133,134,135]. Although not yet confirmed in large studies, these preliminary findings are highly promising. We believe that LAAO might be a valuable alternative to lifelong anticoagulation in advanced CKD patients with AF, thereby providing an effective thromboembolic prevention without increasing the risk of life-threatening bleeding events. The main drawback of endocardial LAAO is the risk of possible thrombus formation on the occlusion device. Several antithrombotic strategies have been empirically adopted in clinical practice to avoid this worrisome complication [126,127,136,137]. To date, the most common approach is based on the use of aspirin, initially with clopidogrel and then alone, to prevent activation of platelets coming in contact with the atrial surface of the device until complete endothelialization is achieved [131,132,133,134,135]. Randomized clinical trials are needed to identify the best antithrombotic therapy to prevent device-related thrombosis and explore the efficacy of LAAO in high-risk populations with a reduced safety margin between stroke prevention and bleeding risk (e.g., end-stage CKD, elderly).

## 7. Conclusions

Patients with CKD, especially with ESRD already in RRT, represent a challenging population for the choice of long-term anticoagulant therapy; however, mounting evidence suggests that DOACs might be a better alternative than warfarin as a result of the lower incidence of AKI and WRN and a better risk/benefit ratio.

## Figures and Tables

**Table 1:** Stages of CKD according to eGFR. CKD: chronic kidney disease; eGFR: estimated glomerular filtration rate; ESRD: end stage renal disease.

Stage	Description	eGFR (mL/min/1.73m^2^)
1	Normal or High	>90
2	Mildly decrease	60–89
3a	Mildly to moderately decreased	45–59
3b	Moderately to severely decreased	30–44
4	Severely decreased	15–29
5	Renal failure (ESRD)	<15 or dialysis

CDK is defined as either kidney damage or eGFR < 60 mL/min/1.73 m^2^ for ≥3 months.

## Abbreviations

ACEi	Angiotensin converting enzyme inhibitor
AF	Atrial fibrillation
AKI	Acute kidney injury
ARN	Anticoagulant-related nephropathy
CKD	Chronic kidney disease
DM	Diabetes mellitus
DKD	Diabetic kidney disease
DOAC	Direct oral anticoagulant
eGFR	Estimated glomerular filtration rate
ESRD	End stage renal disease
INR	International normalized ratio
LAAO	Left atrial appendage occlusion
MGP	Matrix gamma-carboxyglutamate protein
RRT	Renal replacement therapy
VKA	Vitamin K antagonist
WRN	Warfarin-related nephropathy
